# The potential of anti-malarial compounds derived from African medicinal plants, part II: a pharmacological evaluation of non-alkaloids and non-terpenoids

**DOI:** 10.1186/1475-2875-13-81

**Published:** 2014-03-06

**Authors:** Fidele Ntie-Kang, Pascal Amoa Onguéné, Lydia L Lifongo, Jean Claude Ndom, Wolfgang Sippl, Luc Meva’a Mbaze

**Affiliations:** 1 Chemical and Bioactivity Information Centre, Department of Chemistry, Faculty of Science, University of Buea, PO Box 63, Buea, Cameroon; 2 Department of Pharmaceutical Sciences, Martin-Luther University of Halle-Wittenberg, Wolfgang-Langenbeck Str. 4, 06120 Halle, Saale, Germany; 3 Department of Chemistry, Faculty of Science, University of Douala, PO Box 24157, Douala, Cameroon

**Keywords:** Africa, Malaria, Medicinal plants, Natural products, Traditional medicine

## Abstract

Malaria is currently a public health concern in many countries in the world due to various factors which are not yet under check. Drug discovery projects targeting malaria often resort to natural sources in the search for lead compounds. A survey of the literature has led to a summary of the major findings regarding plant-derived compounds from African flora, which have shown anti-malarial/antiplasmodial activities, tested by *in vitro* and *in vivo* assays. Considerations have been given to compounds with activities ranging from “very active” to “weakly active”, leading to >500 chemical structures, mainly alkaloids, terpenoids, flavonoids, coumarins, phenolics, polyacetylenes, xanthones, quinones, steroids and lignans. However, only the compounds that showed anti-malarial activity, from “very active” to “moderately active”, are discussed in this review.

## Background

Malaria is caused by protozoans of the genus *Plasmodium* (*Plasmodium falciparum*, *Plasmodium malariae*, *Plasmodium ovale*, and *Plasmodium vivax*) [1,2]. According to the World Health Organization (WHO), about half of the world’s population is at risk of malaria and one to two million annual deaths (mostly among African children) can be attributed to malaria alone [3,4]. The causative agent is transmitted by the female *Anopheles* mosquito species, which has also developed resistance against insecticides, such as dichlorodiphenyltrichloroethane (DDT), and chemoprophylaxis has not often yielded the expected results [2]. Additionally, the disease-causing protozoans have developed resistance against most of the drugs currently used to treat malaria. There is an urgent need to discover new active compounds.

Nature, and particularly plants are a potential source of new anti-malarial drugs, since they contain a quantity of metabolites with a great variety of structures and pharmacological activities. Traditional preparations (with the use of macerations, extracts, steam baths, concoctions, and decoctions from plant materials) have been the main source of treatment of malaria in Africa [5] and other continents where the disease is endemic [6,7]. Thus, with failing treatment regimens, many research groups in Africa (African indigenous research groups and their foreign collaborators) have resorted to plant sources in the quest to expand the anti-malarial chemotherapeutic arsenal [1,8,9]. This effort has been motivated by the use of these plant materials in the treatment of malaria and fevers in African traditional medicine (ATM). The results from Africa and other continents have been quite promising and hence there has been a general call for the use of natural products as drugs or as sources of inspiration for the development of novel anti-malarials, in order to possibly avoid problems related to drug resistance [10-12].

It is believed that the next generation anti-malarials or the scaffolds necessary for their synthesis may be found in the plants currently used in ATM [13,14]. However, the last review on anti-malarial compounds from African flora dates back about ten years [13], with other reviews focusing on plant-screening campaigns in particular regions and/or countries in Africa [15-35] or on active compounds obtained by bioassay-guided fractionation efforts from given countries and/or regions, not covering an entire continent [36-41]. Even though natural products that are active against *P. falciparum* have been discussed in a number of review papers [1,42-48], the goal has been to provide an coverage of the most promising anti-malarials from the entire African continent, by giving an overview of the most pertinent *in vitro* and *in vivo* screening results reported in the literature. The most successful anti-malarials in use to date have been derived from natural product sources (quinolones/artemisinins). It is indeed a glaring omission that the African continent, despite its rich ethno-pharmocological heritage, is yet to yield a significant contribution in this respect. Clearly, as a first step, a systematic review of the many traditional therapeutic options is needed and this review addresses an important issue in this aspect. In part I, the most promising alkaloids and terpenoids were presented [49], while in this part the most interesting findings for flavonoids, coumarins, phenolics, polyacetylates, xanthones, quinones, steroids, and lignans are shown. The last part of the work is essentially focused on the cheminformatic analysis of >500 natural products (NPs), derived from African medicinal plants, which have demonstrated from weak to very good *in vitro* anti-malarial activities, with a focus on molecular descriptors related to “drug-likeness”, drug metabolism and pharmacokinetics (DMPK). The predicted properties of plant-derived anti-malarials are those related to drug absorption, distribution, metabolism, elimination, and toxicity (ADMET) based on *in silico* computed molecular descriptors.

### Promising anti-malarial agents derived from African flora

By convention, activities were categorised into “very potent”, “good”, “good to moderate”, “weak”, “very weak” and “inactive”. Following criteria used by Mahmoudi *et al.*[50] and Wilcox *et al.*[51], a pure compound was considered highly active if IC_50_ < 0.06 μM, being active with 0.06 μM ≤ IC_50_ ≤ 5 μM, weakly active when 5 μM ≤ IC_50_ ≤ 10 μM and compounds with IC_50_ > 10 μM were considered inactive. The following inhibition percentages were proposed for *in vivo* activity of antimalarial extracts at a fixed dose of 250 mg kg^−1^ day^−1^: 100-90% (very good activity); 90-50% (good to moderate); 50-10% (moderate to weak); 0% (inactive) [52].

### Flavonoids

**Figure 1:**
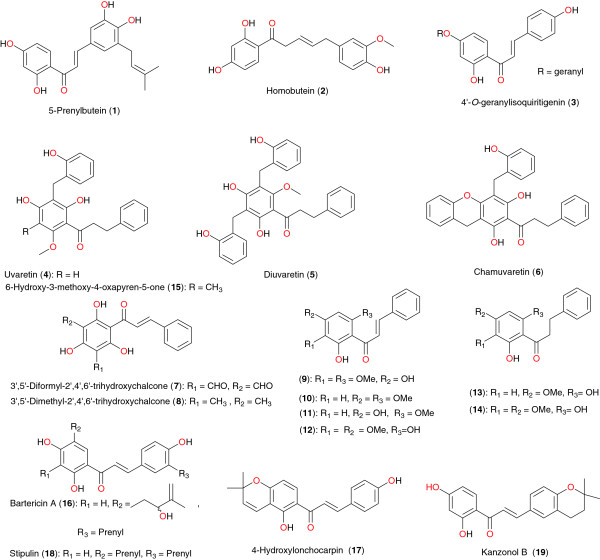
Promising anti-malarial chalcones derived from African flora.

**Figure 2:**
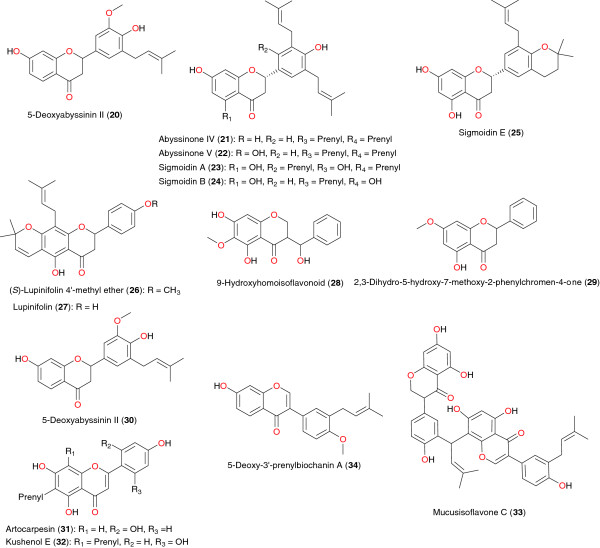
Flavanones and isoflavonoid with anti-plasmodial activity, derived from plants used in African traditional medicine.

**Figure 3:**
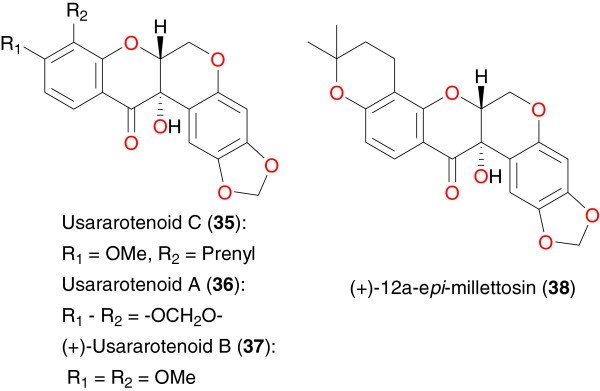
Promising anti-malarial retenoids from African medicinal plants.

Several bioactive flavonoids have been derived from medicinal plants growing in Africa. Even though the molecular mechanism of action of anti-malarial activities of flavonoids is not fully elucidated, it is believed that flavonoids act by inhibiting the fatty acid biosynthesis (FAS II) of the parasite [53,54]. Some flavonoids have also been shown to inhibit the influx of *L*-glutamine and myoinositol into infected erythrocytes [55]. The active anti-malarial flavonoids are summarized in Table 1, while the chemical structures are shown in Figures 1, 2 and 3.

**Table 1:** Summary of promising anti-malarial flavonoids derived from African flora

**Compound subclass**	**Isolated metabolites**	**Plant species (Family)**	**Part of plant studied**	**Place of harvest (locality, country)**	**Author, reference**
Chalcones	**1** and **2**	*Erythrina abyssinica* (Leguminosae)	Stem bark	Thika town, Kenya	Yenesew *et al.*[56,57]
	**3**	*Milletia usaramensis* ssp*. usaramensis* (Leguminosae)	Stem bark	Jadini Forest, Kenya	Yenesew *et al.*[58]
	**4, 5** and **6**	*Uvaria* sp*.* (Annonaceae)	Leaves, stem and root bark	Tanzania	Nkunya *et al.*[59]
	**7** and **8**	*Friesodielsia obovata* (Annonaceae)	Root bark	Tabora district, Tanzania	Joseph *et al.*[60]
	**9, 10, 11, 12, 13,** and **14**	*Polygonum senegalense* (Polygonaceae)	Aerial parts	Nairobi, Kenya	Midowo *et al*. [61]
	**4** and **15**	*Uvaria puguensis* (Annonaceae)	Stem bark	Pugu Forest Reserve, Tanzania	Makangara *et al.*[62]
	**16, 17, 18,** and **19**	*Dorstenia barteri* (Moraceae)	Twigs	Tombel, Cameroon	Ngameni *et al.*[63]
Flavanones	**20, 21, 22, 23, 24,** and **25**	*Erythrina abyssinica* (Leguminosae)	Stem bark	Thika town, Kenya	Yenesew *et al.*[56,57]
	**26** and **27**	*Derris trifoliate* (Leguminosae)	Seed pods	Coast Province, Kenya	Yenesew *et al.*[64]
	**28** and **29**	*Polygonum senegalense* (Polygonaceae)	Aerial parts	Nairobi, Kenya	Midowo *et al*. [61]
	**30**	*Erythrina abyssinica* (Leguminosae)	Stem bark	Thika town, Kenya	Yenesew *et al.*[57]
	**31** and **32**	*Morus mesozygia* (Moraceae)	Stem bark	Centre Province, Cameroon	Zelefack *et al.*[65]
Isoflavones	**33**	*Ficus mucuso* (Moraceae)	Figs	Tongolo-Yaoundé, Cameroon	Bankeu *et al.*[66]
	**34**	*Erythrina sacleuxii* (Leguminosae)	Root bark	Kenya	Andayi *et al.*[67]
Retonoids	**35, 36, 37** and **38**	*Milletia usaramensis* ssp*. usaramensis* (Leguminosae)	Stem bark	Jadini Forest, Kenya	Yenesew *et al.*[58]

### Chalcones

Several anti-malarial flavonoids have been isolated from the stem bark of *Erythrina abyssinica* by Yenesew *et al.*[56,57]. These include chalcones, prenylated and non-prenylated isoflavones and flavones, pterocarpenes, and flavenes. All compounds exhibited moderate anti-malarial activity against the D6 and W2 strains of *P. falciparum.* The ethyl acetate extract of the stem bark of this plant showed anti-plasmodial activity against the chloroquine-sensitive (D6) and chloroquine-resistant (W2) strains of *P. falciparum* with IC_50_ values of 7.9 and 5.3 μg mL^−1^, respectively. From this extract, a new chalcone, 2′,3,4,4′-tetrahydroxy-5-prenylchalcone or 5-prenylbutein (**1**), a new flavanone, 4′,7-dihydroxy-3′-methoxy-5′-prenylflavanone (trivial name, 5- deoxyabyssinin II) and homobutein (**2**), along with known flavonoids have been isolated as the antiplasmodial principles. Yenesew *et al*. also investigated the stem bark of *Milletia usaramensis* ssp. *usaramensis* (Leguminosae) from Kenya [58]. The chalcone 4′-*O*-geranylisoquiritigenin (**3**) was isolated. This compound exhibited moderate to weak antiplasmodial activity against the D6 and W2 strains of *P. falciparum*. Nkunya *et al*. investigated several Tanzanian species of the genus *Uvaria*[59]. Petroleum ether, dichloromethane and methanol extracts of leaves, stem, and root bark of nine *Uvaria* species: *Uvaria dependens*, *Uvaria faulknerae*, *Uvaria kirkii*, *Uvaria leptocladon*, *Uvaria lucida* ssp. *lucida*, *Uvaria* sp. (Pande), *Uvaria scheffleri*, and *Uvaria tanzaniae* were tested for their *in vitro* activity against the multidrug-resistant K-l strain of *P. falciparum*. The IC_50_ values of the extracts varied between 5 and 500 μg mL^−1^. The most active extracts were obtained from the stem and root bark of *U. lucida* ssp. *lucida* and *Uvaria* sp. (Pande) and the root bark of *U. scheffleri*, all of which had IC_50_ values between 5 and 9 μg mL^−1^. The investigations of these authors yielded five important chalcones, uvaretin (**4**), diuvaretin (**5**), triuvaretin, isotriuvaretin and chamuvaretin (**6**). These compounds showed moderate to high antiplasmodial activity against the multidrug-resistant K-1 strain of *P. falciparum*, with respective IC_50_ values of 3.49, 4.20, 46.02, 20.85 and 5.32 μg mL^−1^. Joseph *et al*. also isolated two bioactive chalcones; 3′,5′-diformyl-2′,4′,6′-trihydroxychalcone (**7**) and 3′,5′-dimethyl-2′,4′,6′-trihydroxychalcone (**8**) from the root bark of *Friesodielsia obovata*[60]. These two compounds exhibited moderate antiplasmodial activity against the K-1 strain of *P. falciparum*, with respective IC_50_ values of 23 and 9.7 μg mL^−1^. The same trend of activity was observed against the NF54 strain, against which the compounds had IC_50_ values of 29 and 8.5 μg mL^−1^ respectively.

The new homoisoflavonoid, 5,7-dihydroxy-3-(hydroxy-phenyl-methyl)-6-methoxy-chroman-4-one or polygohomoisoflavanone (**9**) was isolated from the aerial exudates of *Polygonum senegalense*, along with the known chalcones **10** to **14**, by Midowo *et al*. [61]. The new compound, along with other components of the aerial exudate showed good antiplasmodial activities towards D6 and W2 strains of *P. falciparum*. Mukaranga *et al*. investigated the stem bark of *Uvaria puguensis* (Annonaceae) from Tanzania [62]. Repeated chromatography of the petroleum ether and chloroform extracts yielded uvaretin (**4**) and the new phenanthrenoid 6-hydroxy-3-methoxy-4-oxapyren-5-one (**15**), which has been named puguenolide.

The chalcones bartericin A (**16**), and 4-hydroxylonchocarpin (**17**), stipulin (**18**) and kanzonol B (**19**) were isolated from the twigs of *Dorstenia barteri* (Moraceae) from Cameroon by Ngameni *et al*. [63]. These compounds were evaluated in culture against the W2 strain of *P. falciparum*. The evaluated compounds were found to be active *in vitro* against *P. falciparum*, **16**, **17** and **18**, demonstrating particular potencies with relatively low IC_50_ values (2.15 μM, 3.36 μM and 5.13 μM respectively). The observed activities confirmed the chalcones as potential leads for the development of anti-malarials.

### Flavanones

The flavanones 5-deoxyabyssinin II (**20**), abyssinone IV (**21**), abyssinone V (**22**), sigmoidins A (**23**), B (24) and E (**25**), as well as 5-deoxyabyssinin II (**30**) were isolated from the stem bark of *Erythrina abyssinica* (Leguminosae), harvested in Kenya [57]. The investigations of Yenesew *et al*. demonstrated that these compounds exhibited anti-malarial properties against the W2 and D6 strains of *P. falciparum* with IC_50_ values varying from 4.9 to 13.6 μM against the latter strain and from 5.9 to 13.3 μM against the former strain [56,57]. The same authors investigated the seed pods of *Derris trifoliate* (Leguminosae) [64]. From the dichloromethane-methanol (1:1) extract, a new flavanone derivative (*S*)-lupinifolin 4′-methyl ether (**26**) was isolated, in addition to the known flavonoids lupinifolin (**27**) and rotenone. Lupinfolin only showed moderate *in vitro* antiplasmodial activity against the D6 and W2 strains of *P. falciparum*. The different parts of this plant showed larvicidal activities against *Aedes aegypti* and rotenoids were identified as the active principles [64]. Midowo *et al*. examined the aerial exudates of *Polygonum senegalense* and reported the isolation, characterization and antiplasmodial activities of the first 9-hydroxyhomoisoflavonoid (**28**), 2,3-dihydro-5-hydroxy-7-methoxy-2-phenylchromen-4-one (**29**), along with the antiplasmodial activities of some of chalconoids and a flavanone isolated along with it from the surface exudate of *Polygonum senegalense*[61].

The antiplasmodial and cytotoxic activities of flavonoids and arylbenzofuran derivatives from *Morus mesozygia* were investigated by Zelefack *et al*. [65]. This plant is used in treating many diseases, including malaria and fever. Fractionation of the methanolic extract of its stem bark led to the isolation and identification of two flavonoids: artocarpesin (**31**) and kushenol E (**32**), among other compounds (mulberrofuran F, bartericin A and 4-hydroxylonchocarpin). The methanolic extract and the isolated compounds were tested for antiplasmodial activity against the chloroquine-resistant FcB1 *P. falciparum* strain and cytotoxicity on MCF-7 human breast cancer cells. It was found that all compounds were active against the FcB1 strain of *Plasmodium*, with compounds **31**, **32** and mulberrofuran F exhibiting particular potency (with the median inhibitory concentrations IC_50_ = 2.5- 2.6 μg mL^−1^).

### Isoflavones

The isoflavone dimer, mucusisoflavone C (**33**), derived from the figs of *Ficus mucuso*, harvested near Yaoundé in Cameroon, exhibited a weak inhibitory activity against the validated drug target *P. falciparum* enoyl-ACP reductase (*Pf*ENR), with an IC_50_ value of 7.69 μM [66].

The acetone extracts of the root bark and stem bark of *Erythrina sacleuxii* showed antiplasmodial activities against the D6 and W2 strains of *P. falciparum*. Further chromatographic separation of the acetone extract of the root bark by Andayi *et al*. afforded a new isoflavone, 7-hydroxy-4′-methoxy-3′-prenylisoflavone, named 5-deoxy-3′-prenylbiochanin A (**34**) along with known isoflavonoids as the antiplasmodial principles [67]. Flavonoids and isoflavonoids isolated from the stem bark of *E. sacleuxii* were also tested and showed antiplasmodial activities.

### Rotenoids

*Milletia usaramensis* ssp. *usaramensis* is a plant growing in East Africa, which is reported to contain anti-malarial flavonoids, particularly rotenoids [58]. Seven rotenoids have been reported from this species, including usararotenoid C (**35**), usararotenoid A (**36**), (+)-usararotenoid B (**37**), and (+)-12a-*epi*-millettosin (**38**). These compounds exhibited moderate to weak antiplasmodial activity against the D6 and W2 strains of *P. falciparum*. Yenesew *et al*. further established some structure–activity relationships. It was observed that rotenoids containing a prenyl unit or a 2,2-dimethylpyrano substituent were more potent than the non-prenylated rotenoid, e g, usararotenoid A. It was also reported that there is no significant activity for usararotenoid B, suggesting the importance of the carbonyl function at C-12 in usararotenoid A for the weak antiplasmodial activity observed.

### Phenolics

**Figure 4:**
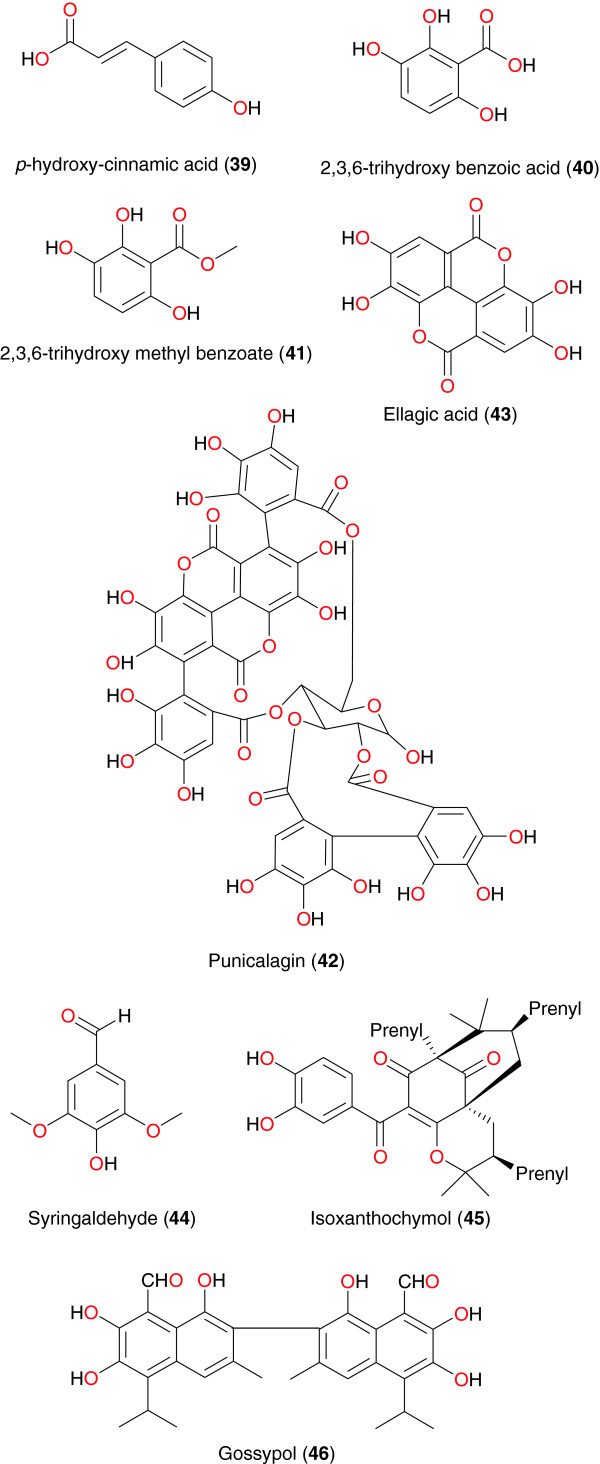
Promising anti-malarial/antiplasmodial phenolics from African medicinal plants.

Zofou *et al*. have isolated *p*-hydroxy-cinnamic acid (**39**), along with other compounds, atranorin, specicoside, 2β,3β,19α-trihydroxy-urs-12-20-en-28-oic acid, from the stem bark of *Kigelia africana* (Bignoniaceae), harvested from Cameroon and performed the drug interactions of the isolated compounds among themselves, as well as their combination effects with quinine and artemether [68]. The antiplasmodial activity and drug interactions were evaluated against the multidrug-resistant W2mef strain of *P. falciparum*. The results equally showed a slight synergistic effect between atranorin and 2β,3β,19α-trihydroxy-urs-12-20-en-28-oic acid (combination index, CI of 0.82) whereas the interaction between specicoside and *p*-hydroxycinnamic acid was instead antagonistic (CI of 2.67). All three compounds were shown to significantly act in synergy with some first line malaria drugs like artemether (CI of 0.42 to 0.71). More excitingly, none of these four compounds showed any significant sign of toxicity against the monkey kidney cell strains LLC-MK2 (selectivity index below 10). Compound **39** exhibited antiplasmodial activitity against the W2mef strain with an IC_50_ value of 2.11 μg mL^−1^[69]. The origins of the isolated anti-malarial/antiplasmodial phenolics are shown in Table 2, while the chemical structures are shown in Figure 4.

**Table 2:** Summary of promising anti-malarial phenolics, polyacetylenes and quinones derived from African flora

**Compound class**	**Isolated metabolites**	**Plant species (Family)**	**Part of plant studied**	**Place of harvest (locality, country)**	**Author, reference**
Phenolics	**39**	*Kigelia africana* (Bignoniaceae)	Stem bark	Bandjoun, Cameroon	Zofou *et al.*[68,69]
	**40** and **41**	*Sorindeia juglandifolia* (Anacardiaceae)	Fruits	Mt. Kalla, Yaoundé, Cameroon	Boyom *et al*. [70]
					Kamkumo *et al*. [71]
	**42**	*Combretum molle* (Combretaceae )	Stem bark	Tigray region, Northern Ethiopia	Asres *et al.*[72]
	**43**	*Alchornea cordifolia* (Euphorbiaceae)	Leaves	Ivory Coast	Banzouzi *et al.*[73]
	**44**	*Vepris uguenensis* (Rutaceae)	Roots	Baringo District, Kenya	Cheplogoi *et al.*[74]
	**45**	*Garcinia polyantha* (Guttiferae)	Roots	Mt Kalla, Yaoundé, Cameroon	Lannang *et al*. [75]
	**46**	*Gossypium* sp*.* (Malvaceae)	Seeds	Diverse regions from the continent	Deck *et al*. [76]
Polyacetylenes	**47, 48, 49** and **50**	*Cussonia zimmermanii* (Araliaceae)	Root bark	Pugu Forest, Tanzania	Senn *et al.*[77]
Quinones	**51, 52, 53** and **54**	*Hoslundia opposita* (Lamiaceae)	Root bark	Tanzania	Achenbach *et al.*[78]
	**55**	*Cassia siamea* (Fabaceae)	Stem bark	Otu (Oyo State), Nigeria	Ajaiyeoba *et al.*[79]
	**56, 57, 58, 59, 60** and **61**	*Psorospermum glaberrimum* (Hypericaceae)	Root bark	Ekombité, Cameroon	Lenta *et al.*[80]
	**62, 63, 64, 65** and **66**	*Harungana madagascariens* (Hypericaceae)	Root bark	Bazou, Cameroon	Lenta *et al.*[81]
	**67**	*Spathodea campanulata* (Bignoniaceae)	Stem bark	Ibadan, Nigeria	Makinde *et al*. [82]
	**68** and **69**	*Kniphophia foliosa* (Asphodelaceae)	Roots		Dagne *et al*. [83]
					Bringmann *et al*. [84]
	**70** and **71**	*Kigelia pinnata* (Bignoniaceae)	Root bark		Weiss *et al*. [85]

In an effort to identify a lead compound for anti-malarial drug discovery, Kamkumo *et al.* investigated the fruits of *Sorindeia juglandifolia* (Anacardiaceae) from Mt Kalla in Cameroon and tested the isolated compounds *in vitro* against the *P. falciparum* W2, against field isolates of *P. falciparum*, and against the *P. falciparum* recombinant cysteine protease falcipain-2 [70,71]. The main end products of the activity-guided fractionation were 2,3,6-trihydroxy benzoic acid (**40**) and 2,3,6-trihydroxy methyl benzoate (**41**). Overall, nine fractions tested against *P. falciparum* W2 and falcipain-2 were active, with IC_50_ values of varying from 2.3 to 11.6 μg mL^−1^ for W2, and 1.1-21.9 μg mL^−1^ for falcipain-2. Purified compounds (**40** and **41**) also showed inhibitory effects against *P. falciparum* W2 (IC_50_s 16.5 μM and 13.0 μM) and falcipain-2 (IC_50_s 35.4 and 6.1 μM). In studies of *P. falciparum* isolates from Cameroon, the plant fractions demonstrated IC_50_ values of 0.14-19.4 μg mL^−1^ and compounds (**40** and **41**) values of 6.3 and 36.1 μM. *In vivo* assessment of compound **40** showed activity against *Plasmodium berghei* strain B, with mean parasitaemia suppressive dose and curative dose of 44.9 mg kg^−1^ and 42.2 mg kg^−1^, respectively. Active fractions were found to be safe in mice after oral administration of 7 g kg^−1^ body weight. These results suggest that further investigation of the anti-malarial activities of natural products from *S. juglandifolia* will be appropriate.

The Ethiopian medicinal plant *Combretum molle* (Combretaceae), reported to possess genuine anti-malarial activity, was investigated by Asres *et al*. [72]. The fractionation of the stem bark extract yielded punicalagin (**42**) as the active compound. This compound exhibited *in vitro* activity against the 3D7 strain of *P. falciparum* with IC_50_ values of 2.19 μg mL^−1^. Ellagic acid (**43**), derived from the leaves of *Alchornea cordifolia* (Euphorbiaceae), showed good activity against *P. berghei* in mice with an ED_50_ in the range of 0.2-0.151 μg mL^−1^[73]. Cheplogoi *et al*. investigated the roots of *Vepris uguenensis* (Rutaceae), harvested from the Baringo District, Kenya [74]. Syringaldehyde (**44**) was identified as an active compound, exhibiting moderate antiplasmodial activity against two strains of *P. falciparum*, with IC_50_ values of 13.0 μg mL^−1^ (chloroquine-susceptible 3D7 strain) and 21.4 μg mL^−1^ (chloroquine-resistant FCM29 strain), respectively.

From the methanol extract of roots of *Garcinia polyantha*, Lannang *et al.* isolated Isoxanthochymol (**45**), which exhibited *in vitro* anti-malarial activity against *P. falciparum* and showed strong chemosuppression of parasitic growth [75]. The compound exhibited anti-malarial activity with an IC_50_ of 2.21 μM. This was lower than the IC_50_ of the other five co-occurring compounds (garcinane, smeathxanthones A and B, chefouxanthone, isoxanthochymol, magnificol, and β-sitosterol and garciniaxanthone I), which ranged from 2.5 to 4.1 μM. The compounds were administered over a period of four days to the culture and the number of parasites was determined daily. Control experiments were performed either without treatment or with administration of 0.032 μM chloroquine in the same solvent. Gossypol (**46**), derived from the seeds of cotton plant (*Gossypium* sp., Malvaceae), exhibits a variety of biological activities, including antispermatogenic, anti-cancer, antiparasitic and antiviral activity. Deck *et al*. demonstrated that this compound also showed anti-malarial activity against both chloroquine-sensitive and chloroquine-resistant strains of *P. falciparum*, with IC_50_ values in the order of 10 μM [76]. The presence of aldehyde functional groups renders gossypol toxic and in the light of this fact, authors further investigated synthetic analogues of compound **46** for biological activity. It was found that the synthetic analogues lost toxicity while retaining antiplasmodial activity.

### Polyacetylenes

**Figure 5:**
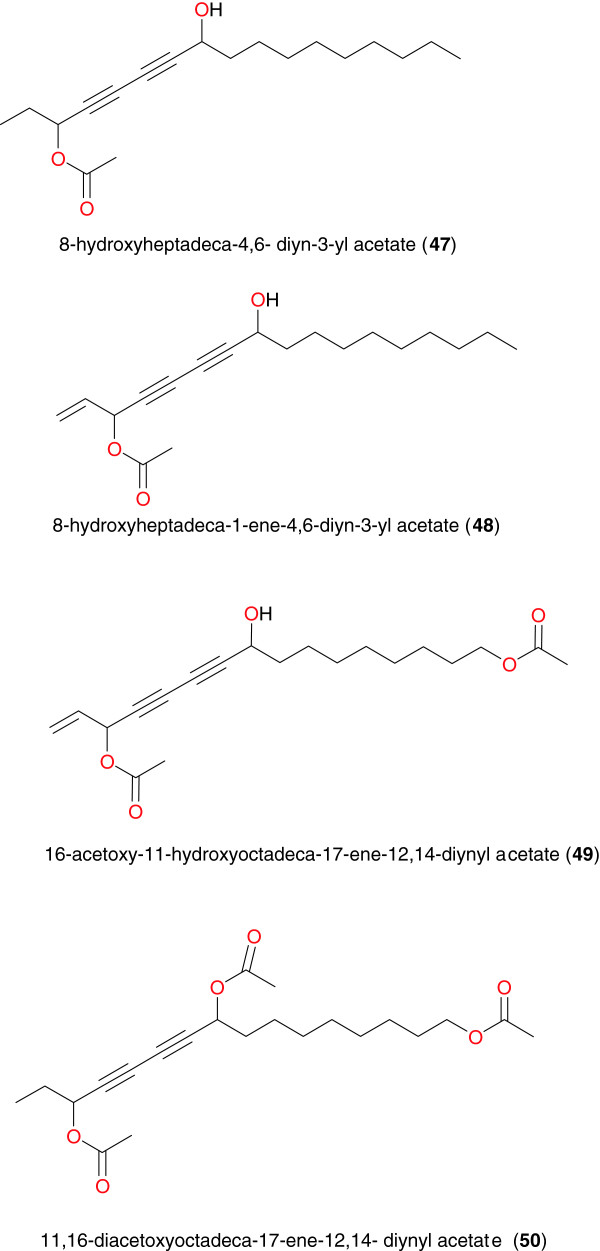
Some anti-malarial/antiplasmodial polyacetylenes from African medicinal plants.

Polyacetylenes have unique chemical structures, which make them rare and often unstable and very reactive. They thus have a wide variety of biochemical and pharmacological uses. Senn *et al*. investigated the root bark extract of *Cussonia zimmermanii* (Araliaceae) from the Pugu Forest in Tanzania, a plant commonly used to treat malaria, fever and epilepsy [77]. Four polyacetylenes were isolated, namely: 8-hydroxyheptadeca-4,6- diyn-3-yl acetate (**47**), 8-hydroxyheptadeca-1-ene-4,6-diyn-3-yl acetate (**48**), 16-acetoxy-11-hydroxyoctadeca-17-ene-12,14-diynyl acetate (**49**) and 11,16-diacetoxyoctadeca-17-ene-12,14-diynyl acetate (**50**), Figure 5. Compounds **47** to **49** showed high anti-malarial activity against *P. falciparum*, with IC_50_ values of 5.9, 0.44 and 0.84 μM respectively.

### Quinones

**Figure 6:**
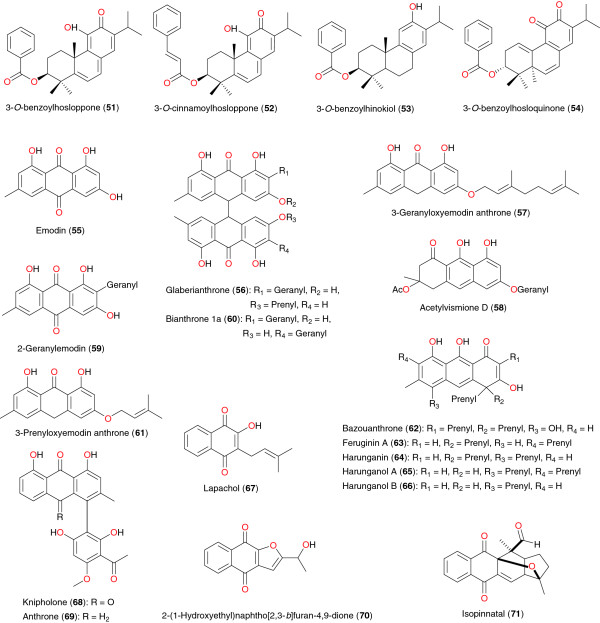
Anti-malarial/antiplasmodial quinones from African medicinal plants.

Quinones also exhibit diverse pharmacological properties, including anti-malarial activity. Four quinones have been isolated from the root bark of *Hoslundia opposita* by Achenbach *et al*. [78], including 3-*O*-benzoylhosloppone (**51**), 3-*O*-cinnamoylhosloppone (**52**), 3-*O*-benzoylhinokiol (**53**), and 3-*O*-benzoylhosloquinone (**54**), Figure 6. The antiplasmodial activities of compound **51** have helped to validate the ethnobotanical use of the plant in the treatment of malaria [78]. The isolation of these compounds was carried out as a result of an ethnomedical use of *H. opposita* in the treatment of malaria. The *n*-hexane extract root bark gave an IC_50_ of 5.6 μg mL^−1^ and also exhibited a 26% inhibition of growth of *P. berghei* in mice, at a daily dose of 190 mg kg^−1^ body weight, for four days [78]. Only compound **51** was tested and showed significant *in vitro* activity against the multidrug-resistant K-1 strain and the chloroquine-sensitive NF54 strain of *P. falciparum*, with IC_50_ values of 0.4 and 0.22 μg mL^−1^, respectively. The other metabolites were not screened due to the limited amount available [78].

*Cassia siamea* (Fabaceae) was identified from southwest Nigerian ethnobotany as a remedy for febrile illness. This led to the bioassay-guided fractionation of stem bark of the plant extract, for assessing the *in vitro* anti-malarial activity. Emodin (**55**) and lupeol were isolated from the ethyl acetate fraction. Both compounds were found to be the active principles responsible for the antiplasmodial property with IC_50_ values of 5 μg mL^−1^ respectively [79].

Six quinones were derived from the root bark extract of *Psorospermum glaberrimum* (Hypericaceae) from Cameroon by Lenta *et al*. [80]. These include glaberianthrone (**56**), 3-geranyloxyemodin anthrone (**57**), acetylvismione D (**58**), 2-geranylemodin (**59**), bianthrone 1a (**60**), and 3-prenyloxyemodin anthrone (**61**). The *n*-hexane extracts and the isolated compounds were tested *in vitro* for their antiplasmodial activity against *P. falciparum* (W2). The *n*-hexane extract showed good antiplasmodial activity, with IC_50_ of 0.87 μg mL^−1^, meanwhile 3-geranyloxyemodin anthrone and acetylvismione D showed the best potencies against *P. falciparum* W2 strain with IC_50_ of 1.68 μM and 0.12 μM, (0.66 μg mL^−1^ and 0.054 μg mL^−1^), respectively. The same authors investigated the root bark of *Harungana madagascariens* (Hypericaceae), a plant whose roots and bark are used by traditional healers to treat malaria in West Province of Cameroon [81]. These authors isolated bazouanthrone (**62**), a new anthrone derivative, along with the known compounds, feruginin A (**63**), harunganin (**64**), harunganol A (**65**), and harunganol B (**66**). In order to validate its ethnobotanical use, the antiplasmodial activity of the isolated compounds were evaluated in culture against W2 strain of *P. falciparum*. All the compounds were found to be active against the *Plasmodium* parasites with bazouanthrone (**62**) showing particular potency (IC_50_ = 1.80 μM).

Makinde *et al*. investigated the action of extracts of the stem bark of *Spathodea campanulata* (Bignoniaceae) from Nigeria on *Plasmodium berghei berghei* in mice [82]. The blood schizontocidal activity of the extracts was studied in early and established infections using chloroquine as the reference drug. The prophylactic action of the extracts was also investigated with pyrimethamine as the standard drug. The hexane and chloroform extracts of the stem bark showed blood schizontocidal action in both the four-day test and Rane test. The chloroform extract demonstrated some prophylactic properties while the aqueous extract did not show any significant anti-malarial property. In addition, these authors were able to identify the active anti-malarial ingrediant to be lapachol (**67**). The other anti-malarial quinones identified were knipholone (**68**) and anthrone (**69**) from *Kniphophia foliosa* (Asphodelaceae) [83,84], as well as 2-(1-hydroxyethyl)naphtho[2,3-*b*]furan-4,9-dione (**70**) and isopinnatal (**71**) from *Kigelia pinnata* (Bignoniaceae) [85]. These compounds were tested against chloroquine-sensitive (T9 − 96) and -resistant (K-1) *P. falciparum* strains and for cytotoxicity using KB cells. Compound **70** possessed good activity against both strains [IC_50_ values 627 nM (K1), 718 nM (T9 − 96)]. Isopinnatal (**71**) and the co-occurring kigelinol and isokigelinol exhibited lower activity against both strains. Bringmann *et al*. also reported that knipholone (**68**) and three of its natural derivatives from the same plant, as well as seven structurally related but simplified compounds, have been examined for their antiplasmodial activity against asexual erythrocytic stages of two strains of *P. falciparum in vitro* (K1/chloroquine-resistant and NF 54/chloroquine-sensitive) [84]. All the phenylanthraquinones showed considerable activity, with only little cytotoxicity, while their anthraquinone and phenyl moieties were completely inactive.

### Coumarins

**Figure 7:**
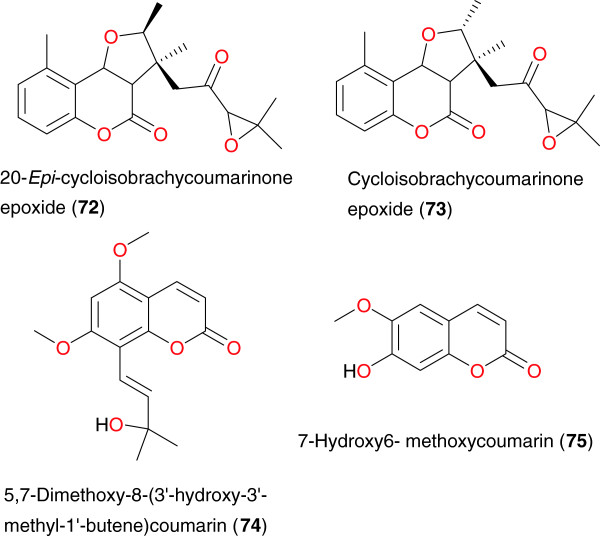
Coumarins from from African medicinal plants with promising anti-malarial/antiplasmodial activities.

Anti-malarial coumarins have been identified by Cubukcu *et al*. [86] and by Noster *et al*. [87] from *Vernonia brachycalyx* (Asteraceae) and *Toddalia asiatica* (Rutaceae), respectively. Cubukcu *et al*. identified two isomeric 5-methylcoumarins from the roots of *V. brachycalyx*; 20-*epi*-cycloisobrachycoumarinone epoxide (**72**) and cycloisobrachycoumarinone epoxide (**73**), Table 3 and Figure 7. The results of the antiplasmodial assays against the chloroquine-susceptible 3D7 and chloroquine- resistant Dd2 strains of *P. falciparum*, showed that compound **72** was weakly active, with IC_50_ values of 160 μM and 54 μM, while for compound **73**, the IC_50_ values were 111 μM and 54 μM, respectively. Noster *et al*. also isolated compound **73** from the ether extract of *Exostema caribaeum* but however only moderate activity [87]. In addition, Oketch-Rabah *et al*. isolated a new anti-malarial coumarin, 5,7-dimethoxy-8-(30-hydroxy-30-methyl- 10-butene) coumarin (**74**), from the roots of *Toddalia asiatica*[87]. This compound showed moderate activity against the chloroquine-sensitive K39 and chloroquine-resistant V1/S strains of *P. falciparum* strains, with IC_50_ values of 16.2 μg mL^−1^ and 8.8 μg mL^−1^, respectively.

**Table 3:** Summary of promising anti-malarial coumarins and xanthone derived from African flora

**Compound class**	**Isolated metabolites**	**Plant species (Family)**	**Part of plant studied**	**Place of harvest (City, Country)**	**Author, Reference**
Coumarins	**72** and **73**	*Vernonia brachycalyx* (Asteraceae)	Roots		Cubukcu *et al.*[86]
	**74**	*Toddalia asiatica* (Rutaceae)	Roots	Rachuonyo District, Kenya	Oketch-Rabah *et al*. [88]
	**75**	*Schefflera umbellifera* (Araliaceae)	Leaves	Limpopo, South Africa	Mthembu et al. [89]
Xanthones	**76** and **77**	*Hypericum lanceolatum* (Hypericaceae)	Stem bark	Mt. Bamboutos, Cameroon	Zofou *et al*. [90]
	**78, 79, 80, 81, 82** and **83**	*Allanblackia monticola* (Guttiferae)	Stem bark	Bagangté, Cameroon	Azebaze *et al*. [91]
	**84, 85** and **86**	*Symphonia globulifera* (Clusiaceae)	Seeds	Fundong, Cameroon	Ngouela *et al*. [92]
	**87, 88, 89** and **90**	*Pentadesma butyracea* (Guttiferae)	Fruit pericarp	Bazou, Cameroon	Lenta *et al*. [93]

The anti-malarial coumarin 7-hydroxy6-methoxycoumarin or scopoletin (**75**) was isolated from the dichloromethane leaf extract of *Schefflera umbellifera* (Araliaceae), harvested from Limpopo, South Africa by Mthembu *et al*. [89]. This compound was evaluated *in vitro* against both the chloroquine-susceptible (D10) and chloroquine-resistant (K-1) strains of *P. falciparum* for anti-malarial activity, with an IC_50_ value of 28.2 μg mL^−1^.

### Xanthones

**Figure 8:**
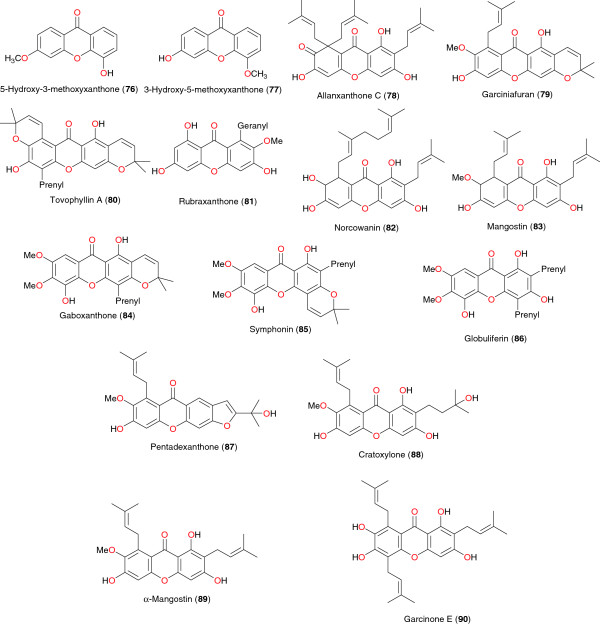
Xanthones from from African medicinal plants with promising anti-malarial/antiplasmodial activities.

The anti-malarial xanthones; 5-hydroxy-3- methoxyxanthone (**76**) and 3-hydroxy-5-methoxyxanthone (**77**) were isolated from stem bark of *Hypericum lanceolatum* (Hypericaceae) from Cameroon by Zofou *et al*. [90], with IC_50_ values of 13.56 μg mL^−1^, and 8.28 μg mL^−1^, respectively, on the multidrug-resistant W2mef strain of *P. falciparum*. Six other anti-malarial xanthones were isolated from the methanol extract of the stem bark of *Allanblackia monticola* (Guttiferae) from Cameroon, by Azebaze *et al*. [91]. These included allanxanthone C (**78**), garciniafuran (**79**), tovophyllin A (**80**), rubraxanthone (**81**), norcowanin (**82**) and mangostin (**83**), Figure 8. Allanxanthone C exhibited an IC_50_ of 1.3 μM on FcM29 and an IC_50_ of 6.9 μM on F32. The molecules with interesting activities are known to be norcowanin (IC_50_ of 6.3 μM on F32) and mangostin (IC_50_ of 4.1 μM on FcM29 and IC_50_ of 7.8 μM on F32 [91]. More interestingly, these molecules showed no significant toxicity against the human melanoma cell A375 cell-line. The antiplasmodial activities of xanthones isolated from the seed shells of *Symphonia globulifera* were reported against the W2 *Plasmodium* sp. with respective IC_50_ values of 3.53, 1.29 and 3.17 μM, for gaboxanthone (**84**), symphonin (**85**) and globuliferin (**86**) [92]. Bioassay-guided fractionation of the fruit pericarp of *Pentadesma butyracea*, using the antiplasmodial test, led to the isolation of a new bioactive xanthone, named pentadexanthone (**87**) (IC_50_ = 3 μM against W2), together with three known compounds: cratoxylone (**88**) (IC_50_ = 2.89 μM), α-mangostin (89) (IC_50_ = 2.77 μM), and garcinone E (90) (IC_50_ = 0.41 μM) [93].

### Steroids

**Figure 9:**
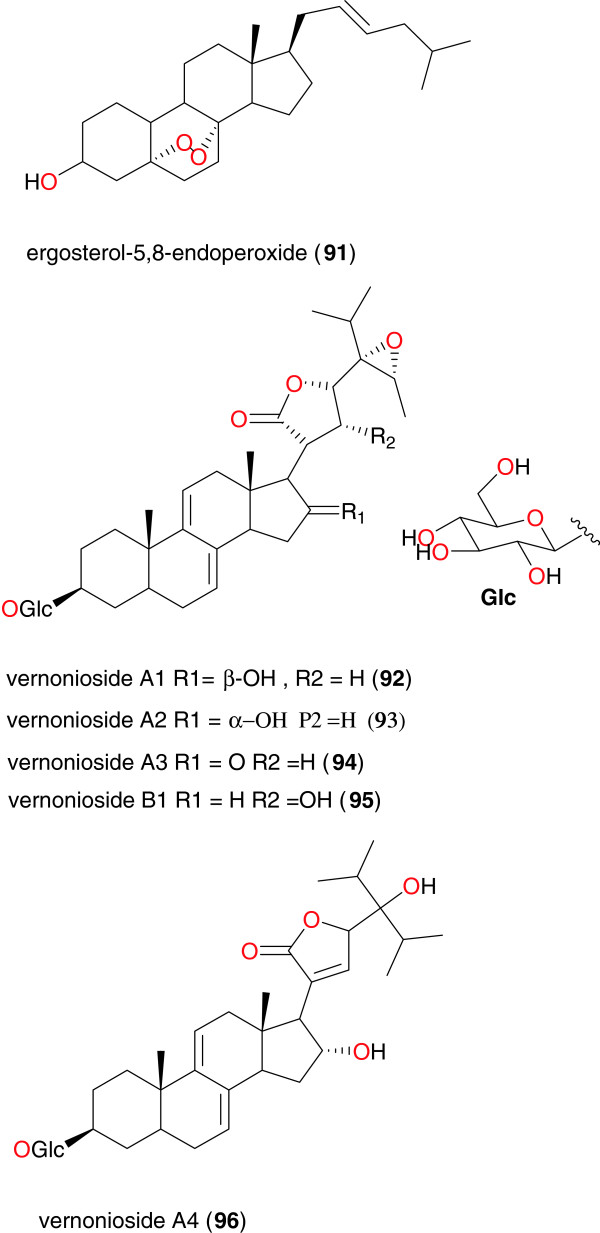
Steroids from African medicinal plants with antiplasmodial activity.

The steroid, ergosterol-5,8-endoperoxide (**91**), isolated from the aerial parts of *Ajuga remota*, exhibited high antiplasmodial activity against the chloroquine-sensitive FCA 20/GHA strain of *P. falciparum*, with an IC_50_ value of 8.2 μM [94]. Steroidal saponins with anti-malarial activity have also been isolated from the leaves of *Vernonia amygdalina*[95]. Ohigashi *et al*. reported the isolation of vernonioside A1 (**92**), A2 (**93**), A3 (**94**), A4 (**95**) and B1 (**96**), Figure 9 and Table 4. These saponins had weak antiplasmodial activity against the multidrug-resistant K-1 strain of *P. falciparum*, with IC_50_ values of 139.7, 94.1, 245.1, 81.8 and 46.1 μg mL^−1^, respectively [95]. These saponins are also reported to be the bitter compounds in the leaves of *V. amygdalina*.

**Table 4:** Summary of promising anti-malarial steroids, lignans, other antiplasmodial compounds derived from African flora

**Compound sub class**	**Isolated metabolites**	**Plant species (Family)**	**Part of plant studied**	**Place of harvest (City, Country)**	**Author, Reference**
Steroids	**91**	*Ajuga remota* (Lamiaceae)	Aerial parts	Nairobi, Kenya	Kuria *et al*. [94]
	**92, 93, 94, 95** and **96**	*Vernonia amygdalina* (Asteraceae)	Young pith of trees	Mahale Mt. National Park, Tanzania	Ohigashi et al. [95]
Lignanes	**97, 98, 99, 100, 101, 102** and **103**	*Pycnanthus angolensis* (Myristicaceae)	Stem bark	São Tomé and Príncipe islands	Ramalhete *et al*. [96]
	**104**	*Asparagus africanus* (Asparagaceae)	Roots	Kenya	Oketch-Rabah *et al*. [97]
Others	**105**	*Lippia javanica* (Verbenaceae)	Leaves and stalks	Limpopo, South Africa	Ludere *et al*. [98]
	**106**	*Helichrysum cymosum* (Asteraceae)	Whole plant	South Africa	Jakupovic *et al*. [99]
					Vuuren *et al.*[100]
	**107, 108, 109** and **110**	*Vernonia staehelinoides* (Asteraceae)	Leaves	Magaliesburg, South Africa	Pillay *et al*. [101]
	**111**	*Hypericum lanceolatum* (Hypericaceae)	Stem bark	Mt. Bamboutos, Cameroon	Zofou *et al*. [90]
	**112**	*Symphonia globulifera* (Clusiaceae)	Seeds	Fundong, Cameroon	Ngouela *et al*. [92]
	**113**	*Morus mesozygia* (Moraceae)	Stem bark	Centre Province, Cameroon	Zelefack *et al.*[61]
	**114** and **115**	*Kigelia africana* (Bignoniaceae)	Stem bark	Bandjoun, Cameroon	Zofou *et al.*[64,65]
	**116** and **117**	*Glossocalyx brevipes* (Monimiaceae)	Leaves	Kumba, Cameroon	Mbah *et al*. [102]
	**118**	*Asparagus africanus* (Asparagaceae)	Roots	Kenya	Oketch-Rabah *et al*. [97]
	**119**	*Dracaena mannii and Dracaena arborea* (Dracaenaceae)	Seed pulp	Nigeria	Okunji *et al*. [103]

### Lignans

**Figure 10:**
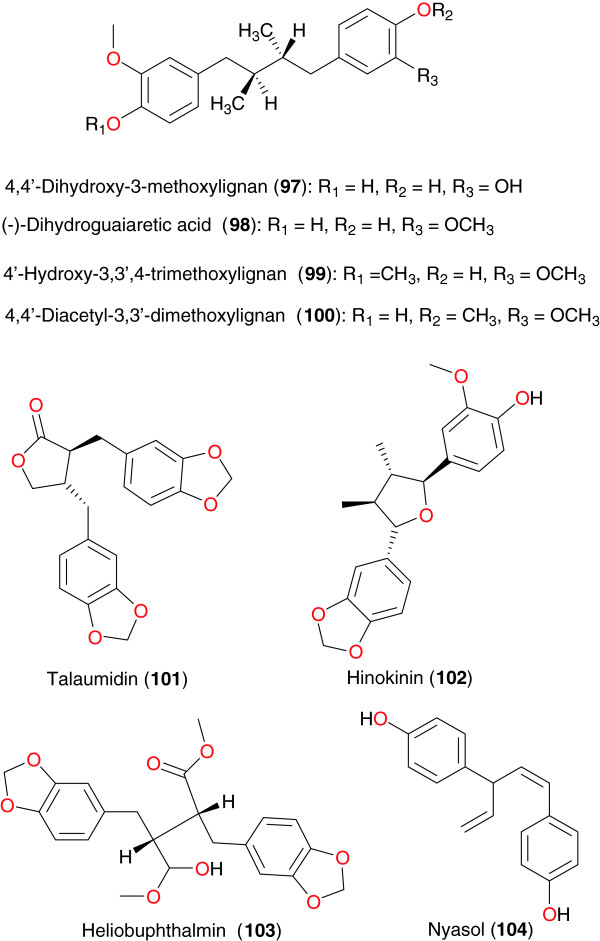
Lignans from African medicinal plants with promising anti-malarial/antiplasmodial activities.

*Pycnanthus angolensis* (Myristicaceae) is a plant used in traditional medicine against several diseases. Its bark has been used to treat fever and malaria in São Tomé and Príncipe islands. Ramalhete *et al*. submitted the dichloromethane extract of the bark to anti-malarial screening and observed an activity against 3D7 *P. falciparum* strain (IC_50_ = 1.6 μg mL^−1^) [96]. This was further subjected to chromatographic bioguided fractionation, yielding the lignans 4,4′-dihydroxy-3-methoxylignan (**97**), (−)-dihydroguaiaretic acid (**98**), 4′-hydroxy-3,3′,4-trimethoxylignan (**99**), 4,4′-diacetyl-3,3′-dimethoxylignan (**100**), talaumidin (**101**), hinokinin (**102**), and heliobuphthalmin (**103**), Figure 10, along with the labdane diterpene ozic acid and the steroids stigmast-4-en-6β-ol-3-one, stigmasterol and β-sitosterol. Furthermore, other compounds were obtained by derivatization. The *in vitro* anti-malarial activity of the compounds was evaluated against 3D7 and Dd2 *P. falciparum* strains. The best *in vitro* activities were exhibited by compound **97** against the 3D7 strain (IC_50_ = 31.0 μg mL^−1^) and by compound **101** against the Dd2 strain (IC_50_ = 20.7 μg mL^−1^).

**Figure 11:**
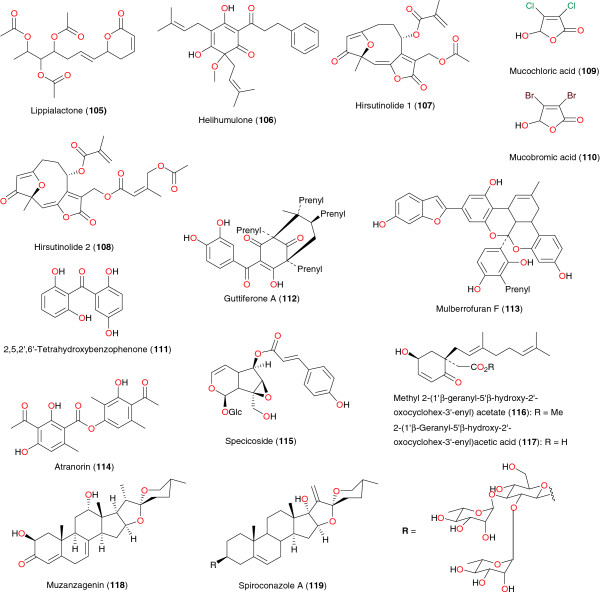
Other metabolites from African medicinal plants with promising anti-malarial/antiplasmodial activities.

*Asparagus africanus* (Asparagaceae) is used by the Akamba tribe in Kenya to treat malaria. A bioassay-guided fractionation of the root extract led to the isolation of the lignan nyasol (**104**), along with the sapogenin muzanzagenin (**119**), Figure 11, as the bioactive compounds responsible for the anti-malarial activity of this plant [97]. Nyasol moderately inhibited *P. falciparum* schizonts with the IC_50_ of 49 μM, while muzanzagenin showed a moderate *in vitro* activity against four different malaria schizont strains the IC_50_ values were 16, 163, 23, and 16 μM, respectively.

### Others

Lippialactone (**105**), derived from the ethyl acetate extract of aerial parts of *Lippia javanicais*, harvested from South Africa, was shown to be active against the chloroquine-sensitive D10 strain of *P. falciparum* with an IC_50_ value of 9.1 μg mL^−1^, and is also mildly cytotoxic [98]. Helihumulone (**106**) was derived from extracts of the whole plant of *Helichrysum cymosum* (Asteraceae) from South Africa by Jakupovic *et al.*[99] and Vuuren *et al.*[100].

The dichloromethane extract of the leaves of *Vernonia staehelinoides* (Asteraceae) showed *in vitro* activity (IC_50_ ~ 3 μg mL^-1^) against the chloroquine-sensitive D10 and the chloroquine-resistant (K-1) strains of *P. falciparum*[101]. Pillay *et al*. further investigated the extract by bioassay-guided fractionation and two structurally related hirsutinolides displaying *in vitro* antiplasmodial activity (IC_50_ ~ 0.2 μg mL^−1^ against D10) were isolated. These were 8α-(2- methylacryloyloxy)-3-oxo-1-desoxy-1,2-dehydrohirsutinolide-13-*O*-acetate (**107**), and 8α- (5′-acetoxysenecioyloxy)-3-oxo-1-desoxy-1,2-dehydrohirsutinolide-13-*O*-acetate (**108**). These were found to be cytotoxic to mammalian Chinese hamster ovarian (CHO) cells at similar concentrations, but proved to be attractive scaffolds for structure-activity relationship studies. Two main privileged substructures, a 2(5*H*)-furanone unit and a dihydrofuran-4- one unit, were identified as potential pharmacophores, which may be responsible for the observed biological activity. Mucochloric and mucobromic acids were selected as appropriate 2(5*H*)-furanone substructures and these were shown to have comparable activity against the D10 and superior activity against the K1 strains relative to the hirsutinolide natural product. Mucochloric and mucobromic acids (**109** and **110**) also showed selective cytotoxicity to the malaria parasites compared to mammalian (CHO) cells *in vitro*. The antiplasmodial data obtained with respect to these two acids suggest that the 2(5*H*)- furanone substructure is a key pharmacophore in the observed antiplasmodial activity. The identification of antiplasmodial hirsutinolides from *V. staehelinoides* suggests that they may play a role in the medicinal properties of the plant, but their potential for the development of anti-malarial drugs is limited due to inherent cytotoxicity and lack of selectivity. The results did however lead to the identification of potential pharmacophores, a 2(5*H*)-furanone unit and a dihydrofuran-4-one unit.

The benzophenone 2,5,2′,6′-tetrahydroxybenzophenone (**111**), from the stem bark of *Hypericum lanceolatum* (Hypericaceae), exhibited an interesting activity against the multidrug-resistant strain W2mef, with an IC_50_ of 13.56 μg mL^−1^[90]. Ngouela *et al*. isolated guttiferone A (**112**) from the seeds of *Symphonia globulifera* (Clusiaceae) [92]. This compound exhibited activity against the W2 *Plasmodium* sp. with IC_50_ of 3.17 μM. Mulberrofuran F (**113**), from the stem bark of *Morus mesozygia* (Moraceae), was active against FcB1-Columbia strain, considered to be resistant against chloroquine, with IC_50_ of 2.6 μg mL^−1^[65]. Atranorin (**114**) and specicoside (**115**), derived from the stem bark extract of *Kigelia africana* (Bignoniaceae) [68,69], were both active against the multidrug-resistant W2mef strain of *P. falciparum* with respective IC_50_ values of 0.67 μg mL^−1^ and 0.52 μg mL^−1^.

The homogentisic acid derivatives methyl 2-(1′β-geranyl-5′β-hydroxy-2′- oxocyclohex-3′-enyl) acetate (**116**) and 2-(1′β-geranyl-5′β-hydroxy-2′- oxocyclohex-3′-enyl) acetic acid (**117**) were isolated by Mbah *et al*. from the leaves of *Glossocalyx brevipes* (Monimiaceae) [102]. Compounds **116** and **117** exhibited both anti-malarial [102] and antisalmonellal activities [104]. The sapogenin muzanzagenin (**118**) and the saponin spiroconazole A (**119**), respectively isolated from *Asparagus africanus* (Asparagaceae) [97] and from the West Africa ‘soap tree’ *Dracaena* sp. (Dracaenaceae) [103], also demonstrated significant anti-malarial activities. Spiroconazole A is reported to exhibit pronounced antileishmanial, anti-malarial and molluscicidal activities.

## Conclusions

In this review, an attempt has been made to summarise the main finding of several research groups engaged in the search for naturally occurring active principles from African medicinal plants against *P. falciparum*. With multiple resistance developed by the malaria parasite, the cry has been towards obtaining new effective drugs. Attempts to develop ‘green pharmacies’ for improved phytomedicines against malaria are being encouraged by some NGOs and governments as part of their efforts to control malaria [105]. Additionally, modern hit/lead discovery efforts for specific anti-malarial drug targets are being encouraged. The trend has been towards accelerating this process by employing computer-based methods such as docking, virtual screening, pharmacophore modelling and binding-free energy calculations for hit/lead identification and combinatorial design of novel inhibitors against known anti-malarial drug targets. The practice of virtual screening is beginning to occupy the centre of drug discovery efforts [106] and it has been verified that developing NP libraries containing readily available compounds for screening virtual hits could be highly useful [107]. The authors of this paper have been developing NP databases containing three-dimensional structures of compounds derived from plants used in ATM [108-110] and using computed molecular descriptors to attempt to predict the pharmacokinetic profiles of NPs [110-112]. Since the role of NPs in drug discovery cannot be overemphasized [111-116], efforts are aimed at providing tools for research groups engaged in anti-malarial drug discovery, beginning with NPs derived from African medicinal plants. This is aimed at cutting down the cost of drug discovery when computational and ‘wet lab’ approaches are combined [117,118]. The intention is to make the current collection of three-dimensional structures of naturally occurring anti-malarials from African medicinal plants available for virtual screening. This shall be the scope of part III of this series.

## Abbreviations

ADMET: Absorption, distribution, metabolism, excretion and toxicity; ATM: African traditional medicine; DMPK: Drug metabolism and pharmacokinetics; FAS II: Fatty acid sytem II; NP: Natural product; WHO: World Health Organization; WM: Western medicine.

## Competing interests

The authors declare that they have no competing interests.

## Authors’ contributions

FNK, LLL, JCN, and LMM conceived the idea. FNK, LLL and PAO participated in data collection. FNK and PAO contributed to data analysis, discussion of results and the conception of the paper under the supervision of LMM, WS, LLL, and JCN. FNK and PAO wrote the first draft of the paper and all authors agreed on the final version before submission.
